# Decoupled morphodynamic responses of dune crests and localized scour across the sand-gravel transition in straight alluvial channels

**DOI:** 10.1371/journal.pone.0339732

**Published:** 2026-09-02

**Authors:** Mustafa Tunc

**Affiliations:** Department of Civil Engineering, Faculty of Engineering, University of Firat, Elazig, Turkey; SRM Institute of Science and Technology: SRM Institute of Science and Technology (Deemed to be University), INDIA

## Abstract

This study empirically and statistically investigates the morphodynamic responses of median sediment grain diameter (*d*_50_) on local erosion and the morphodynamic regime within an alluvial channel. Experiments conducted across three distinct sediment sizes demonstrated a strong positive correlation between flow rate (*Q*) and bedform geometry, specifically maximum scour depth (*d*_*scour*_) and dune crest height (*h*_*dune*_). Crucially, median grain size governed bed stability. The finest sediment exhibited high sensitivity, forming dynamic and regular dunes, while the coarsest sediment showed resistance consistent with armor layer formation and yielded a stable bed geometry. The medium sediment defined a transitional regime of localized scour and bars. High-accuracy empirical models were developed to predict maximum scour depth (R^2^ = 0.97, where R^2^ is the coefficient of determination) and dune crest height (R^2^ = 0.93) as functions of flow rate and median grain diameter. Absolute Relative Deviation (ARD) analysis confirmed the models’ robust reliability (±10–15% error range). These findings provide essential quantitative equations and enhanced insights for the sustainable design and maintenance of hydraulic structures in alluvial environments.

## Introduction

Alluvial based open channels are dynamic and complex natural systems whose morphology, hydraulics, and ecological integrity are constantly changing as a natural result of the complex interaction between fluid dynamics and mobile sediment [1,2]. The continuous interaction between flowing water and erodible boundaries triggers a wide range of events, including sediment transport, local erosion, deposition, and the formation of various bedforms such as ripples, dunes, and antidunes. A comprehensive understanding of these processes is critical for numerous engineering and environmental applications, including sustainable river management, effective flood control strategies, safe navigation, and the resilient design of hydraulic structures [3], [4].

The formation and evolution of bedforms are direct results of the dynamic equilibrium established between the energy of flowing water and the mobility of bed sediments. These morphological features are fundamental to understanding river dynamics, bed resistance, and the ecological health of rivers [5]. Ripples are typically small, asymmetrical forms that develop in fine sands at low flow velocities. Dunes, on the other hand, are larger and three dimensional structures formed in coarser sands and gravels under higher flow regimes, with longer wavelengths and greater dimensions than ripples [6,7]. Inverted dunes, which are in phase with the water surface, can cause significant energy dissipation and instability under supercritical flow conditions.

Local erosion refers to the concentrated erosion of bed material around obstacles in the flow field (bridge piers, sill dunes, or other hydraulic structures) [8,9]. This localized erosion is primarily triggered by the formation of horseshoe vortex systems that intensify increased turbulence, flow accelerations, and shear stresses at specific points. Accurate estimation of scour depth and extent is of great importance for the design and long term safety of hydraulic infrastructure, as excessive scour can compromise structural integrity and lead to catastrophic failures [10].

Despite significant research on the geometric and hydraulic factors affecting local erosion, the undeniable importance of bed material properties, particularly grain size, is increasingly being recognized. Larger grain sizes generally exhibit greater resistance to erosion, reducing maximum erosion depth and affecting the time scale required for the erosion pit to reach equilibrium [11,12]. Conversely, finer, less cohesive sediments are more prone to rapid and deep erosion [13,14]. Recent experimental studies confirm that both sediment properties and their arrangement significantly influence maximum scour depth and scour volume [15,16]. Additionally, it has been observed that the coarsening of bed materials over time, from their initial average diameter, affects foot destabilization due to local scouring [17,18]. The integration of coarse sediment transport into the scour development process, even if previously accumulated, is highlighted as a critical aspect that has not always been fully addressed in the existing literature [19,20]. The use of innovative materials such as nanomaterials with cohesionless soil has also shown promising results in reducing scour depth around structures, further highlighting the complex relationship between bed material composition and scour reduction [21,22].

To contextualize the experimental data within the wider continuum of fluvial processes, the observed bed states are systematically evaluated against the bedform stability criteria for the sand-gravel transition dynamics and the localized flow separation frameworks [23–25]. By aligning our quantitative ranges with historical datasets, this study establishes an explicit link between localized boundary-layer measurements and established large-scale empirical trends, providing a clear sense of the functional range of transitional bedforms. Total boundary shear stress is partitioned into grain roughness and macro-scale form roughness [26–28]. As bedforms evolve, form roughness dominates the hydraulic resistance, altering the local velocity profiles and driving the distinct morphodynamics of the trough zone [29,30]. The generalized assumption that coarser sediments inherently produce larger bedforms is strictly bounded by specific depth-discharge conditions. Mapping the experimental parameters onto the bedform stability diagrams of Van den Berg and Van Gelder (1993) confirms that the observed configurations remain within the stable lower-regime dune phase [31].

The spontaneous formation of bedforms in flowing rivers is a result of the dynamic interaction between the bed and the flow field. Initially, when the water flow is strong enough to move sediment particles on a flat sediment bed, bedforms are formed [32–34]. The underlying principle of these formation mechanisms is the adaptation of the flow area to the initial random disturbance of the seabed and the resulting enhancement of the initial disturbance by gradients in sediment flow [35,36]. The presence of channel forms, particularly geometric features such as sandbar height and velocity, plays a critical role in estimating channel load transport rates [37,38].

The bed forms encountered in streams follow a specific sequence depending on the increase in flow intensity (velocity) [39,40]. Geometric features such as dune height and length can significantly affect water levels, create navigation difficulties, and increase flood risk. Washed out Dunes/Transition: As flow velocity increases further, dunes decrease in size and become less distinct. This transition regime represents the stage at which dunes begin to transform into a flat bed. Flat Bed (Plane Bed); As flow velocity increases further, bed forms completely disappear, and the bed becomes a flat surface. In this case, flow resistance depends solely on particle roughness (skin friction). Anti-dunes are bed forms that occur under supercritical flow conditions (Fr > 1), where water surface waves and bed waves are in the same phase [41,42].

In alluvial channels, energy dissipation occurs as the flow mobilizes solid particles, leading to significant changes in hydrodynamic properties such as the velocity profile near the bed, turbulence intensity, and shear stress distribution. These changes become more pronounced in direct proportion to the amount of sediment in motion [43–45]. When the flow velocity exceeds the critical dune that initiates sediment movement and the flow regime remains in the lower flow regime, the initially flat bed surface gives way to distinct ripple patterns [46,47]. These wave patterns may resemble the morphological patterns created by wind on sandy surfaces or by waves on coastal areas, but their formation mechanisms and dynamics differ in terms of the type of fluid and the interacting forces [48,49].

Yalin (1964) proposed approximate relationships for the wavelengths of different bed formations. According to this, in ripple type waves, the wavelength (*L*) depends on the grain size (*d*) and can be expressed approximately as *L*≈1000*d*. At dunes, the wavelength (*L*) depends on the current depth (*y*) and can be represented by the approximate formula *L*≈5*y* [50]. The boundary layer transitions from hydraulically smooth to hydraulically rough states depending on the ratio of grain size to the viscous sublayer thickness [51]. Rather than behaving like localized scour around fixed engineering structures, subaqueous bedforms co-evolve morphodynamically with the flow field. The destabilization of the dune foot is primarily driven by flow separation and subsequent shear layer turbulence at the lee side, rather than fixed horseshoe vortex systems [52].

While previous studies have addressed generalized bedform dynamics under uniform sediment conditions, a fundamental research gap remains regarding how bed material granulometry controls the decoupled, partially-dependent evolution of dune crests and troughs across the sand-gravel transition boundary. Existing empirical models often over-generalize bedform geometry by treating crest height and scour depth as coupled parameters, which fails in non-uniform or transitional grain-size regimes. To address this gap, the central research question of this investigation is: How does median grain diameter (*d*_50_) govern the independent physical response of dune crests and localized scour holes under uniform subcritical flows? The novelty of this study lies in systematically isolating the quantitative effect of *d*_50_ across the sand-gravel threshold, providing refined empirical formulations that explicitly account for the distinct boundary-layer mechanics governing trough scour and crest stability.

## Materials and methods

**Fig 1:**
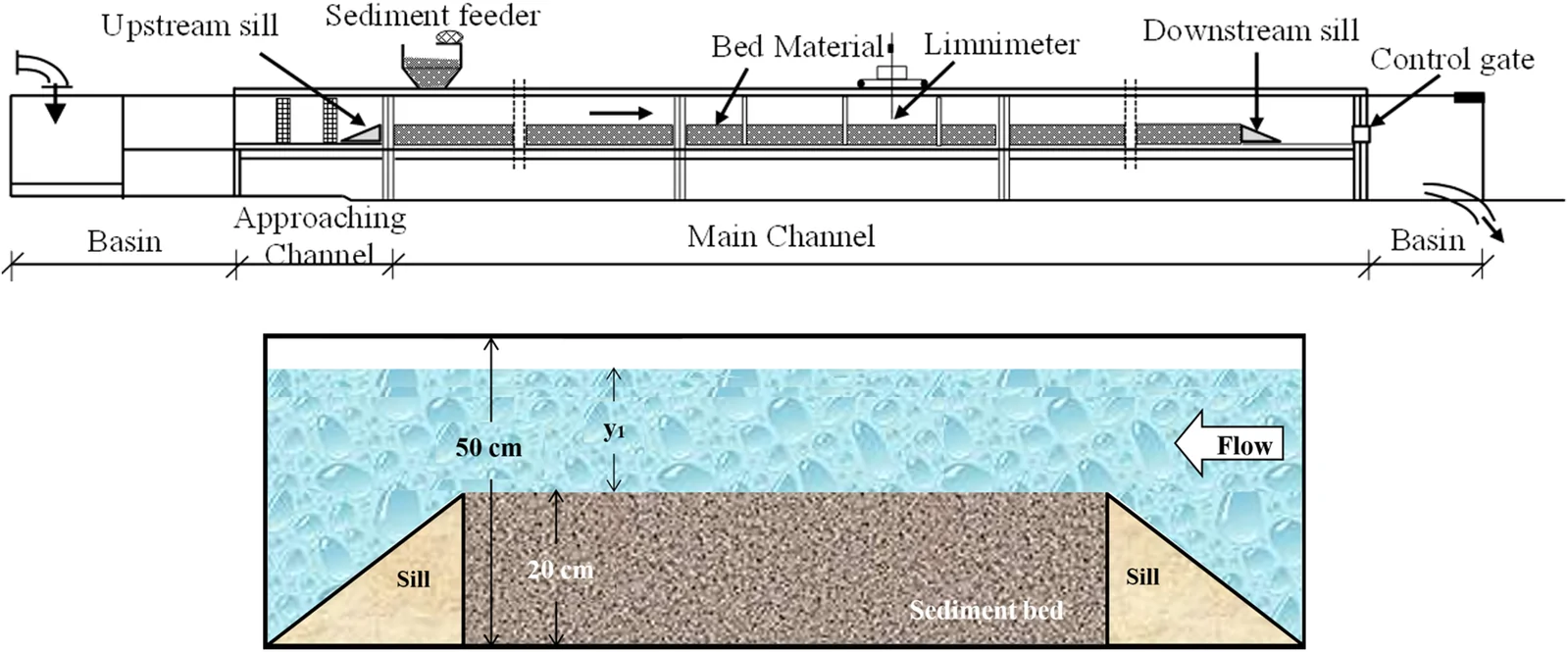
Front view of the experimental setup and experimental system application setup.

The experimental program was executed utilizing the existing laboratory flume setup, which features a fully linear, rectangular cross-section channel designed for precise open-channel flow monitoring. The total width of the channel is 100 cm, of which 50 cm forms the main channel and the remaining 50 cm forms the collection channel. The depth of the main channel is 50 cm, while the depth of the collection channel is 70 cm (Fig 1). The base slope of the main channel is set at 0.1%. The experiments were conducted in a rectangular cross-section linear channel. The study was performed under steady flow conditions, free overflow conditions, and moving bed erosion (*V*_1_/*V*_*c*_ > 1) conditions.

Two ramps, each 20 cm high, have been placed at the upstream and downstream ends of the main channel, as shown in Fig 1. Quartz sand was spread between these dunes to form a sediment bed. The average grain size (*d*_*50*_) of the spread sand was varied to be 1.3 mm, 3.6 mm, and 6.1 mm. The sections before the first sill and after the second sill are supported by sheet metal plates extending to the channel bed, which has an approximate slope of 15°. This arrangement was made to prevent the sand bed from being disturbed. To ensure steady flow conditions (i.e., to ensure flow conditions independent of time), perforated bricks were placed at the upstream end of the channel and at the end of the collection channel. This measure is critical to ensure accurate measurements.

The experimental program was executed in a straight, linear flume with a rectangular cross-section. Fixed artificial ramps (20 cm high) were installed at the upstream and downstream boundaries to stabilize the transition zones. Given that the sediment mixtures span the very coarse sand to fine gravel boundary (*d*_50_ = 1.3 to 6.1 mm), the initial bed stages were monitored to account for the development of transitional bedload sheets and armoring layers [51,53].

The selection of the three distinct median grain diameters (*d*_50_=1.3 mm, 3.6 mm, and 6.1 mm) was carefully determined to isolate and represent fundamentally different transport dynamics and boundary layer conditions within open channel hydraulics. The fine-grained sediment (*d*_50_=1.3 mm) represents a highly mobile boundary where the critical shear stress is easily exceeded under the selected discharge range, promoting the rapid development of classical lower-regime bedforms (ripples and dunes). The intermediate size (*d*_50_=3.6 mm) serves as a transitional operational boundary, characterizing mixed bed behavior where local scour and macro-scale bar formations alternate. Finally, the coarsest sediment (*d*_50_=6.1 mm) was selected to simulate non-cohesive gravel beds operating near or below the threshold of motion, facilitating the empirical investigation of structural bed stability and armor layer formation. This wide distribution enables a comprehensive validation of the proposed empirical models across multiple morphodynamic regimes.

This experimental study was conducted for side weirs centrally located on a linear channel. The base material was spread approximately 4 m backward and 4 m forward from the center of the side weir, covering a total channel section of 8 m. A schematic representation of the experimental system is shown in Fig 1.

Before each experiment, the sand base was thoroughly mixed, compacted, and carefully leveled. The leveling process was performed using a special apparatus mounted on a cart that moved along rails. This method ensured that the sand was spread evenly and at the same level across the entire test area. After the channel bed was compacted and leveled, the flow was initiated in a controlled manner. The valves were slowly opened to gradually introduce water into the channel. As the water slowly rose from the ramp in front of the threshold at the upstream end of the channel and flowed over the sand bed, a second threshold 20 cm high was placed on top of the threshold at the downstream end. This additional threshold reached a total height of 40 cm from the channel bed, preventing the flat form of the sand bed from being disturbed during this critical initial phase.

The water depth in the section of the main channel after the second threshold was allowed to reach the same level as the water depth in the main channel. After the water level was equalized throughout the entire channel, the target flow rate was achieved, and the second threshold above the threshold on the downstream side was slowly raised to prevent disruption of the sand bed form, and the experiment was started. During the experiment, the flow rate was kept constant, and the flow height (*y*_1_) in the channel was adjusted to the desired level using the radial gates located at the end of the channel.

After the experiment period was completed (within a maximum of 15 hours), the valves were slowly closed to preserve the base topography. At this time, the 20 cm high threshold was repositioned on the threshold at the mouth of the linear channel to ensure controlled drainage of water from the channel. Following all these procedures, the maximum scour depth (*d*_*scour*_) and dune height (*h*_*dune*_) formed in the side channel area were precisely measured using a digital limnimeter. Additionally, bottom level measurements were taken on an 8-meter sediment bed for a detailed analysis of the cross-section.

**Fig 2:**
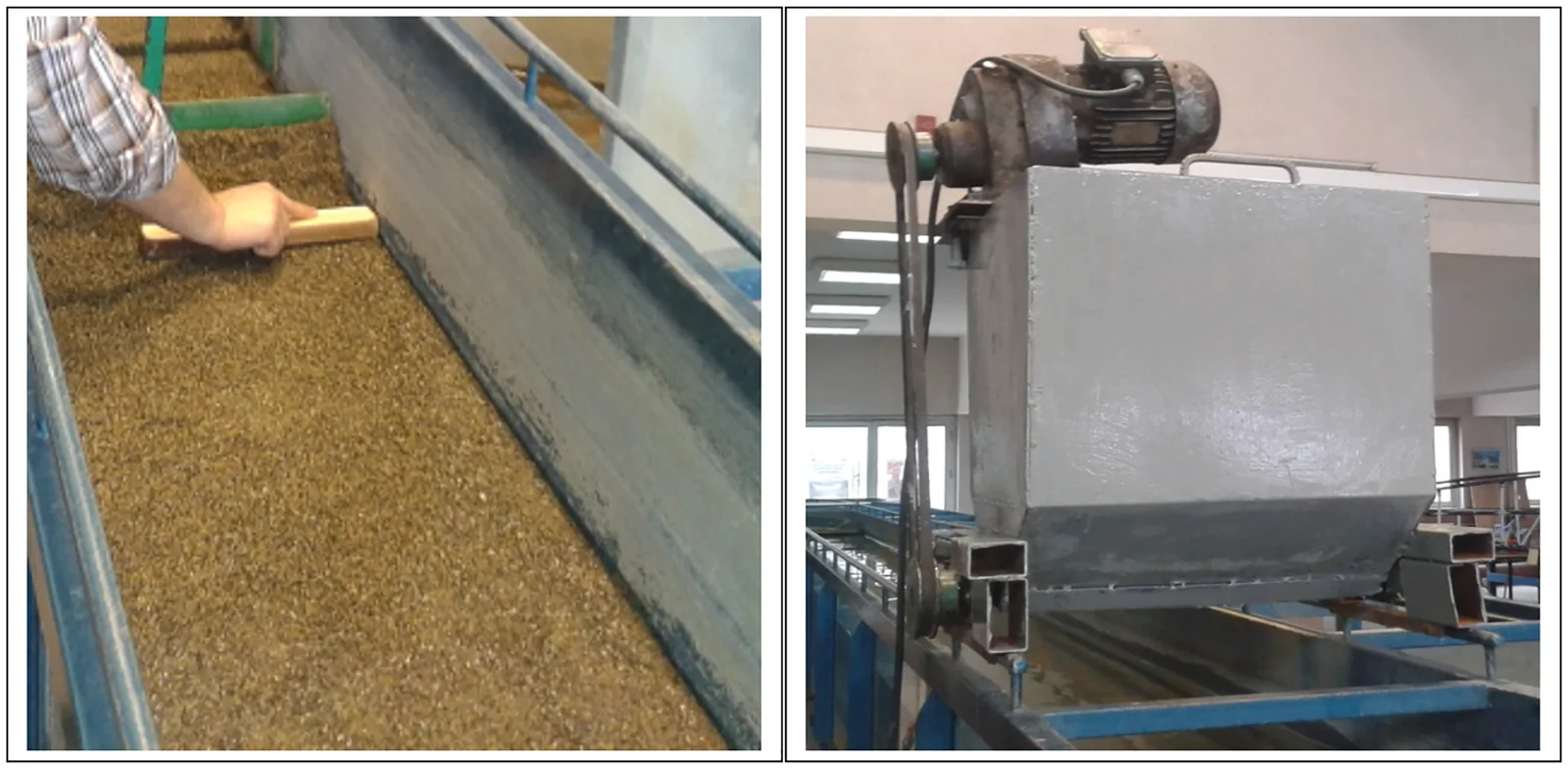
Portable machine adding sediment material to the channel.

Preliminary pilot tests were conducted to determine bed load discharges for various flow conditions. Under moving bed conditions (*V*_1_/*V*_*c*_>1), it was observed that bed waves formed rapidly and the bed remained in constant motion. This indicated that sediment transport occurred at very high levels. The amount of sediment discharged increased continuously with the increase in the *V*_1_/*V*_*c*_ value. Therefore, in order to maintain the moving bed conditions, sediment was continuously added to the channel using a portable machine specially prepared at the Hydraulic Laboratory of the Department of Civil Engineering at Fırat University, as shown in Fig 2. The rate and amount of solid material addition were precisely adjusted based on the *V*_1_/*V*_*c*_ values.

To guarantee highly controlled hydrodynamic conditions, specific mechanical and structural measures were implemented to achieve both steady and uniform flow conditions. Steady flow conditions were maintained by routing the discharge through an upstream stabilization chamber equipped with multiple layers of perforated bricks, which effectively dissipated macroscopic kinetic energy and suppressed initial macro-turbulence before the flow reached the active sediment bed. To establish uniform flow conditions along the 8-meter test section, the channel bed slope was calibrated precisely to a constant longitudinal gradient of 0.1\%. The water surface profile and flow depth (*y*_1_) were monitored continuously at multiple cross-sections using digital limnimeters. Precise adjustments of the downstream radial gates prevented the formation of backwater curves (M1 or M2 profiles), thereby ensuring that the energy grade line remained parallel to the channel bed during steady-state data collection.

Flume discharge and depth-averaged velocity were regulated using an automated electromagnetic flowmeter and tailgate control to maintain uniform flow conditions. The morphological transition zones closely align with the classic dune-bed transition patterns documented by the previous study [54], where the superimposition of smaller bedforms dictates early-stage macro-roughness development.

**Fig 3:**
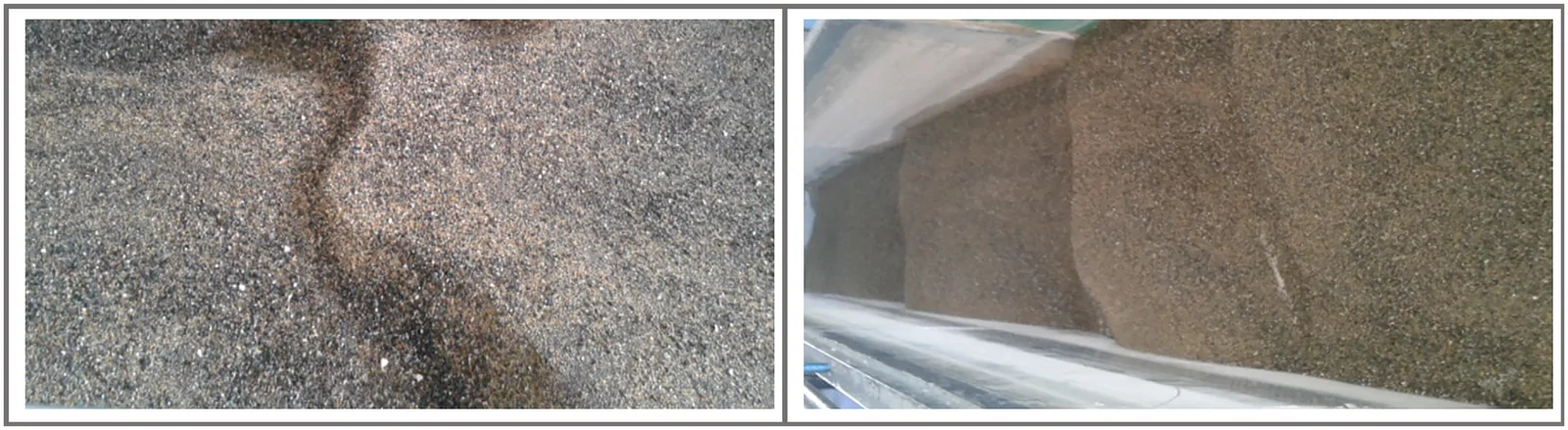
Superimposition of ripples on dunes under subcritical flow conditions.

The experimental observations across the active sediment bed confirmed that under the established subcritical flow conditions (*V*_1_/*V*_*c*_ > 1), the bed configurations remained strictly within the lower-regime dune spectrum. As illustrated in Fig 3, the primary bedforms developed a characteristically asymmetrical longitudinal profile, featuring a gentle stoss slope subjected to continuous tractive sediment movement and a steeper lee slope corresponding to the dynamic friction angle of the submerged sand. Flow separation occurring at the dune crest generated a localized recirculation zone, causing entrained particles to settle into the downstream trough area. This mechanism directly drives the downstream migration of the dunes while maintaining a clear physical decoupling between the maximum crest height and the maximum localized scour depth formed in the trough zone.

To establish a generalized mathematical framework, dimensional analysis was performed using the Buckingham Pi-theorem. The target morphological parameters (dscour and hdune) were formulated as functions of the primary governing hydraulic and physical variables: *f*(*d*_*scour*_, *h*_*dune*_, *Q*, *y*_1_, *V*_1_, *d*_50_, *ρ*, *ρ*_*s*_, *g*, *ν*) = 0, where Q is discharge, *y*_1_ is approach flow depth, *V*_1_ is mean flow velocity, *d*_50_ is median grain diameter, *ρ* is fluid density, *ρ*_*s*_ is sediment density, *g* is gravitational acceleration, and *ν* is kinematic viscosity. Expressing these variables in non-dimensional form yields key governing groups: the approach Froude number ($\text{Fr}=\frac{V_{\text{1}}}{\sqrt{g.y_{\text{1}}}}$) the relative submergence ratio *y*_1_/*d*_50_, and the excess shear stress ratio τ_o_/τ_c_. These non-dimensional parameters were selected based on their fundamental role in characterizing boundary-layer turbulence, flow separation scales, and sediment mobility thresholds.

## Results and discussion

### Implications of scale effects for engineering applications

While the developed empirical models for maximum scour depth (*d*_*scour*_) and dune crest height (*h*_*dune*_) demonstrate high statistical reliability (R^2^ ≥ 0.93) within the laboratory environment, their direct extrapolation to prototype-scale natural rivers requires careful consideration of scaling laws and inherent distortions. Laboratory flume experiments are conventionally designed based on Froude similitude (Fr_model_ = Fr_prototype_). However, it is physically impossible to simultaneously satisfy Reynolds number similitude (Re) and relative sediment roughness scaling (*y*/*d*_50_) without introducing non-linear scaling errors. In real-scale alluvial channels, the water depth-to-grain size ratio (*y*/*d*_50_) is orders of magnitude larger than in laboratory configurations. Furthermore, if sediment grain size were to be scaled down linearly with the geometric scale of the channel, the material would transition into ultra-fine silts or clays, introducing cohesive forces and electrochemical bonds that do not exist in prototype sandy/gravelly rivers. Consequently, the models proposed herein are highly valid for non-cohesive alluvial boundaries operating under rough turbulent regimes. Engineers applying these equations to field designs should introduce appropriate safety factors to accommodate the relative reduction in viscous boundary layer dominance and the macro-scale turbulence structures characteristic of full-scale hydraulic structures.

The structural transition from a highly dynamic mobile bed to a highly stable armored state can be physically interpreted through the dimensionless Shields parameter, *θ*, defined as Equation (1):


$$\begin{array}{c} \theta =\frac{\tau_{o}}{\left(\rho_{s}-\rho \right).g.d_{\text{5}\text{0}}} \end{array}$$ (1)

where *τ*_*o*_ is the bed shear stress, *ρ*_*s*_ is the sediment density, *ρ* is the fluid density, and *g* is the gravitational acceleration.

For the finest sand configuration (*d*_50_=1.3 mm), the mobility ratio *θ*/*θ*_*c*_ (where *θ*_*c*_ is the critical Shields parameter for initial motion) was significantly greater than unity across all tested discharges (*Q*=0.02–0.07 m^3^/s), sustaining a fully generalized transport regime. However, for the coarsest matrix (*d*_50_=6.1 mm), the global Shields parameter hovered near the critical threshold (*θ*≅*θ*_*c*_). Under these constraints, selective sediment transport occurs: the hydrodynamic lift and drag forces are sufficient to entrain only the finer sand fractions present within the interstitial matrix, while the coarser 6.1 mm grains remain stationary. This selective winnowing process drives the progressive development of a geometric armor layer, altering the bed roughness and shielding the underlying sediment layer from further localized erosion.

The physical mechanisms underlying the insensitivity of bedform wavelengths to discharge variations at higher grain sizes (*d*_50_=6.1 mm) stem from the altered allocation of boundary shear stress and grain interlocking dynamics. In finer sediments, an increase in discharge increases the scale of flow separation zones at the dune crests, which directly expands the downstream reattachment length and increases the bedform wavelength (*L*≅5*y*). Conversely, for coarser sediments, the high particle mass limits the saltation height and step length of individual grains. The bed morphology transitions into a structurally rigid configuration governed by particle-to-particle interlocking and high grain roughness (*τ*_*g*_). Because the macro-roughness elements are structurally stable, the flow separation zones become spatially constrained and cannot migrate or expand downstream. Consequently, instead of a hydrodynamic adjustment of the wavelength via active sediment migration, the excess energy of an increased discharge is entirely dissipated through micro-turbulent eddies around the stationary coarse grains, decoupling the bedform wavelength from discharge variations.

### Limitations of the present study

The empirical relationships and morphodynamic findings documented in this study were derived under controlled laboratory conditions (*Q* = 0.02–0.07 m^3^/s), fixed channel width, and three uniform sediment fractions). Consequently, these models should not be interpreted as universal tools directly scalable to all natural alluvial streams. The core novelty of this research lies in its isolation of the median grain diameter (*d*_50_) as a single variable controlling the non-linear morphological transformation from highly mobile lower-regime dunes to stable, armored gravel beds. Future research must expand this parameter space by incorporating varied channel macro-geometries and highly graded sediment mixtures.

In the current experimental study, a series of experiments were conducted for sediment beds with different flow rates (*Q*=0.02–0.07 m^3^/s) and different median particle diameters (*d*_50_=1.3, 3.6, and 6.1 mm). In these experiments, maximum scour depths (*d*_*scour*_) and maximum dune crest heights (*h*_*dune*_) were measured. Numerical methods were developed to estimate *d*_*scour*_ and *h*_*dune*_.

**Fig 4:**
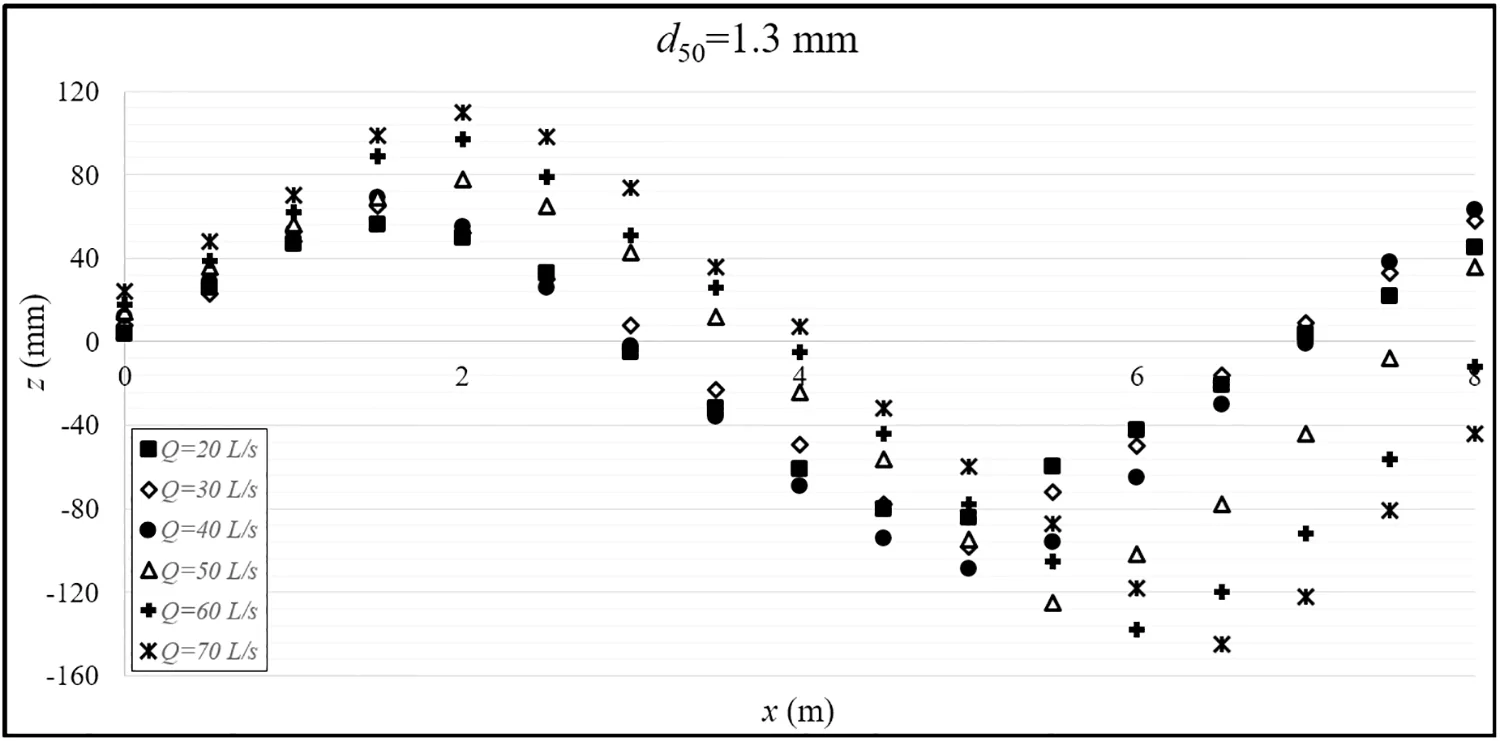
Cross-sectional profiles obtained at different flow rates (*Q*) for a fixed median particle diameter of *d*_50_ = 1.3 mm.

The graph presented in Fig 4 shows the cross-sectional profiles obtained for different discharge (*Q*) values on a sediment bed with a constant median grain size (*d*_50_=1.3 mm). The experiments were conducted along an 8-meter channel covered with sediment. These results clearly demonstrate the effect of increased flow rate on the morphology of the sediment bed. Each symbol in the graph represents a different discharge value. The x-axis (meters) shows the distance along the channel, while the z-axis (mm) shows the vertical height of the bed surface. Positive z values indicate bed elevations (sets/peaks), while negative z values indicate bed depressions (troughs/dissolution areas).

For all flow rates, it is observed that the sediment bed does not form a straight line but has a wavy morphology. This wavy structure indicates the presence of sediment transport and bed formations (e.g., sand ridges or ripples) formed under the flow. These formations result from the shear stress exerted by the flow on the sediment and its transport capacity. For the lowest flow rate value (*Q*=0.02–0.07 m^3^/s), the bed profile shows less undulation compared to other flow rates. Changes in bed height (z values) remain within a smaller range. This indicates that low flow energy is insufficient to rearrange the sediment on a large scale but is sufficient to create small-scale bed formations.

As the flow rate increases to 0.03, 0.04, and 0.05 m^3^/s, an increase in the amplitude and distinctness of the fluctuations in the bed profile is observed. Particularly in areas beyond 2 meters, more pronounced ridges and depressions form on the bed. This indicates that with increasing flow rate, the flow velocity and consequently the shear stress on the sediment increase, leading to the transport of more sediment and the formation of larger-scale bed formations. When the flow rate reaches 0.06 m^3^/s, the bed profile exhibits its most pronounced undulating structure. For this flow rate, the positive and negative z values are spread over the widest range. This indicates that the flow energy maximizes sediment movement, creating large sediment ridges and deep depressions. This may be an indication that the bed formations created within the “lower flow regime” have reached their peak.

For the highest flow rate value of 0.07 m^3^/s, the bed profile shows similar amplitude fluctuations compared to 0.06 m^3^/s, but the positions of the peaks and troughs have shifted slightly in some areas. This suggests that the dynamism and mobility of bed formations increase at very high flow rates, but this increase may not always lead to a proportional increase in amplitude; instead, it may cause changes in the shape and position of the formations.

The graph clearly demonstrates the dependence of sediment transport on flow rate. As flow rate increases, both the amplitude and likely the wavelength of bed formations increase. This is a process that begins when the flow velocity exceeds the critical sediment transport velocity and intensifies with increasing flow rate. The observed bed ripples likely represent sand dunes or sand ripples formed within the lower flow regime. These formations are structural features that move upstream and reshape the bed depending on the direction of flow energy. The formation of distinct peaks at around x=2 m and x=6 m, and a distinct trough at around x=4 m, indicates that the flow dynamics and sediment deposition/transport balance are different in these regions. This may be due to the structure of the experimental channel or the flow developing differently in these regions. While traditional frameworks often treat subaqueous dunes as single geometric units, this study explicitly decouples the morphodynamics of the lee-side trough (scour) from the crest height, as they respond to distinct localized fluid-boundary mechanics.

**Fig 5:**
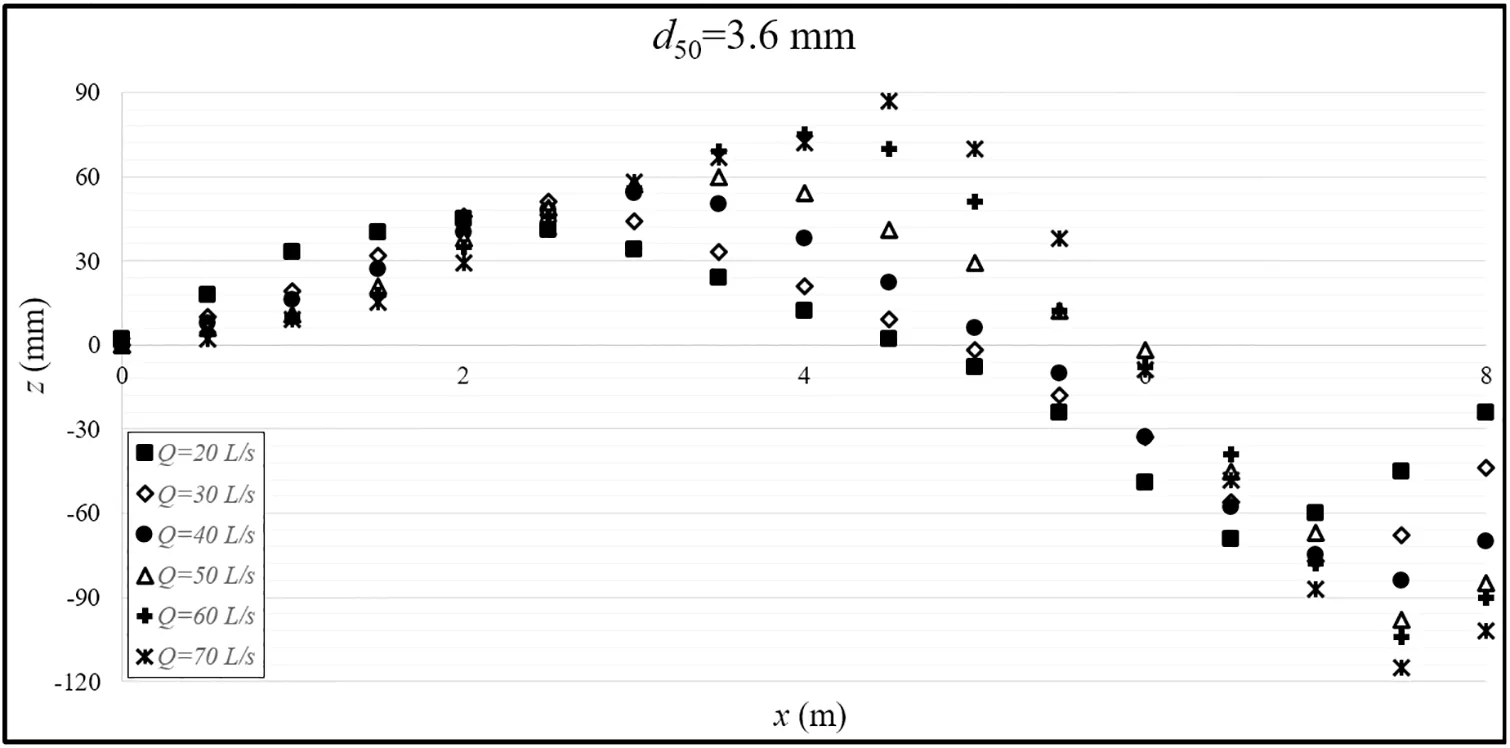
Cross-sectional profiles obtained at different flow rates (*Q*) for a fixed median particle diameter of *d*_50_ = 3.6 mm.

The graph presented in Fig 5 shows the cross-sectional profiles obtained for the same flow rate range (*Q*=0.02–0.07 m^3^/s) in a sediment bed with a larger median grain size (*d*_50_=3.6 mm) compared to the previous series of experiments. These experiments highlight the critical role of grain size in sediment dynamics. In the graph, the x-axis represents the distance along the channel (meters), while the z-axis represents the vertical height of the bed surface (mm). Compared to the previous graph with *d*_50_=1.3 mm, noticeable differences are observed in the bed profiles in this graph. In particular, the structure of the bed surface ripples and their relationship with discharge reflect characteristics specific to the behavior of coarser sediment.

In the *d*_50_=1.3 mm graph, a wavy and periodic bed morphology is observed for all discharges, whereas in this graph (*d*_50_=3.6 mm), the bed profiles are more irregular and characterized by local elevations and depressions rather than a distinct wavy structure. In particular, a general upward trend in the bed is noticeable in the x=0−2 m range, local peaks in the x=2−6 m range, and a deepening scour hole beyond 6 m. At the lowest tested discharge (*Q*=0.02 m^3^/s), the bed profile is relatively smoother, but some sedimentation begins after 6 meters. This indicates that moving larger particles requires more flow energy and that at low discharges, local sediment transport can only occur in areas where flow velocity increases or turbulence intensifies.

In the flow rate range of 0.03–0.05 m^3^/s, more pronounced ridges and depressions are observed in the bed profile. Specifically, there is an uplift (bar formation) around x=4 m and a distinct scouring around x=6 m. This indicates that the increased flow energy is sufficient to displace and rearrange coarse-grained sediments. However, instead of regular wave-like bed formations as seen in the *d*_50_=1.3 mm graph, the initial stages of transition to mixed-form bedforms or a plane bed regime are observed. The bed profile shows the most dramatic change at the highest discharges (*Q*=0.06 m^3^/s and *Q*=0.07 m^3^/s). The crest at x=4 m reaches its highest point, while the scour region beyond x=6 m becomes the deepest. This suggests that the increased flow velocity, due to the larger grain size, tends to form large-scale bars and local scour holes rather than bringing the flow closer to a “plane bed” regime. This observation is consistent with the theory that coarser-grained sediments exhibit different bed formations in different hydrodynamic regimes compared to finer-grained sediments.

This graph shows that coarser-grained sediment exhibits more dune-controlled behavior under flow conditions compared to finer-grained sediment. Since higher shear stress is required to mobilize coarse grains, very little movement is observed at low flows, but as flow increases, large-scale erosion and deposition zones can suddenly form. The morphology in the graph can be associated with coarse-grained bars and the scour mechanisms around them, rather than sand dunes formed in the lower flow regime. These formations typically occur in areas where the flow diverges or where the bed is turbulent. The deep scouring between x=6−8 m, as the experimental channel approaches its end, indicates that the increase in flow velocity and turbulence eroded the bed by intensively transporting sediment.

**Fig 6:**
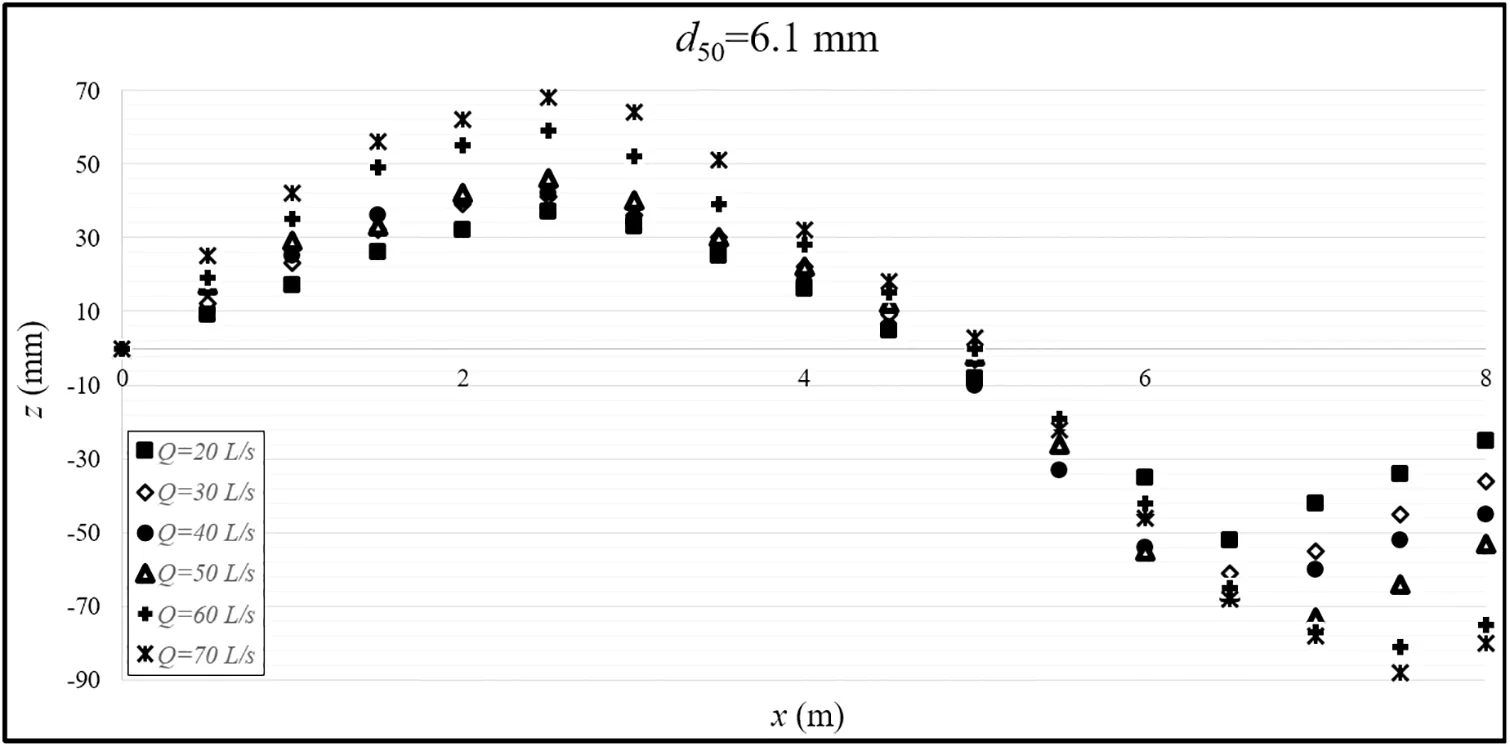
Cross-sectional profiles obtained at different flow rates (*Q*) for a fixed median particle diameter of *d*_50_ = 6.1 mm.

The graph presented in Fig 6 shows the cross-sectional profiles obtained for the coarsest sediment grains in the series (*d*_50_=6.1 mm) under different flow rates (*Q*=0.02-0.07 m^3^/s). This data set demonstrates how river morphology changes radically with increasing grain size. The x-axis (meters) and z-axis (mm) of the graph represent the distance along the channel and the bed level, respectively. Compared to the previous two graphs (Figs 4 and 5), the morphology in this graph exhibits different behavior as a result of coarser sediment, resistance to flow, and a tendency toward immobility. This leads to significant changes in the geometry of bed formations and their response to flow rate.

Unlike the wavy or localized formations observed in sediments with *d*_50_=1.3 mm and *d*_50_=3.6 mm, the bed profile for sediment with *d*_50_=6.1 mm indicates a large-scale, persistent structure that is relatively less affected by flow rate changes. The main morphological features (a peak at approximately 2.5-3.0 m and a trough after 6.0 m) maintain a similar position and general shape for all flow values. This suggests that the flow energy is not sufficient to fundamentally alter the basic structure of this coarse-grained sediment bed, but only to move the smaller-scale grains on its surface.

As the flow rate increases, a slight increase in the width of the bed profile is observed. For example, when the flow rate increases from *Q*=0.02 m^3^/s to *Q*=0.07 m^3^/s, the height of the peak and the depth of the trough increase. However, this increase is not as pronounced as the dramatic changes in sediment size observed earlier. This indicates that even at high flow velocities, most of the sediment remains in the immobile bed, and only the particles in the uppermost layer reach the threshold for movement. At the highest flow rates (*Q*=0.06 m^3^/s and *Q*=0.07 m^3^/s), the peak and trough reaching their maximum values indicates a situation where the flow energy can finally mobilize more particles, but this movement cannot alter the fundamental architecture of the bed formation.

The graph strongly supports the relationship between sediment movement and particle size. The movement threshold (critical shear stress) for this coarse sediment is so high that even at the highest discharge rates, the bed cannot become completely mobile. The observed morphology may be a relict morphology, a permanent sedimentary structure likely determined by the initial conditions of the experimental channel, or large-scale bars formed by the flow's directing action. The flow over the bed is controlled by this persistent structure, meaning that the flow itself is shaped by the bed topography, while the bed topography changes very little in response to the flow. This situation may also indicate the formation of an “armor layer.” At the highest discharges, smaller particles are transported by the flow, while the largest particles remain behind, forming an armor layer that protects the bed from erosion.

These three graphs (Figs 4–6) present the results of experiments conducted in a laboratory setting on sediment beds with different median particle sizes (*d*_50_=1.3 mm, 3.6 mm, and 6.1 mm) under varying flow rates. These data clearly demonstrate the decisive role of sediment particle size in the formation of bed morphology and its response to flow rate changes. In the finest sediment (*d*_50_=1.3 mm), an increase in flow rate increases the amplitude of distinct, regular, and periodic fluctuations in bed profiles. This indicates the bed's high sensitivity to flow energy and the formation of dynamic bed features such as sand ridges or ripples, typically occurring under “low flow regimes.” This bed responds most rapidly and dramatically to changes in flow velocity. In medium-sized sediment (*d*_50_=3.6 mm), the bed morphology has a less regular and periodic structure. Flow increases create local elevations (bars) and deepening scour holes rather than regular ripples across the bed. This morphology reflects a higher threshold requirement for coarser sediment to be mobilized and likely represents a transition state to mixed-form beds or a flat-bed regime. In the coarsest sediment (*d*_50_=6.1 mm), the effect of changes in flow discharge on the bed profile is most limited.

The bed maintains its general geometry (a crest and a trough) independently of discharge changes. Increased flow creates localized rises (bars) and deepening scour holes rather than regular fluctuations across the entire bed. This morphology reflects a higher threshold requirement for the movement of coarser-grained sediment and likely represents a transition state to mixed-form beds or a flat-bed regime. In the coarsest sediment (*d*_50_=6.1 mm), the effect of changes in flow velocity on the bed profile is most limited. The bed maintains its general geometry (a crest and a trough) independently of flow changes. This situation indicates that the threshold for large grains to move is very high and that the flow, rather than changing the basic structure of the bed, only moves the smaller grains on its surface, creating a “armor layer,” or that the bed already exhibits a permanent structure. This bed has the most stable bed structure, responding the least to flow changes.

The strong correlation between scour depth and bedform height must be interpreted relative to the collapse threshold of the dune phase. Unlike the high-flow bimodality and flickering behavior identified by the previous study [47], the transitional beds in this study exhibit a stable, partially-dependent scaling prior to reaching the upper stage plane bed boundary.

**Fig 7:**
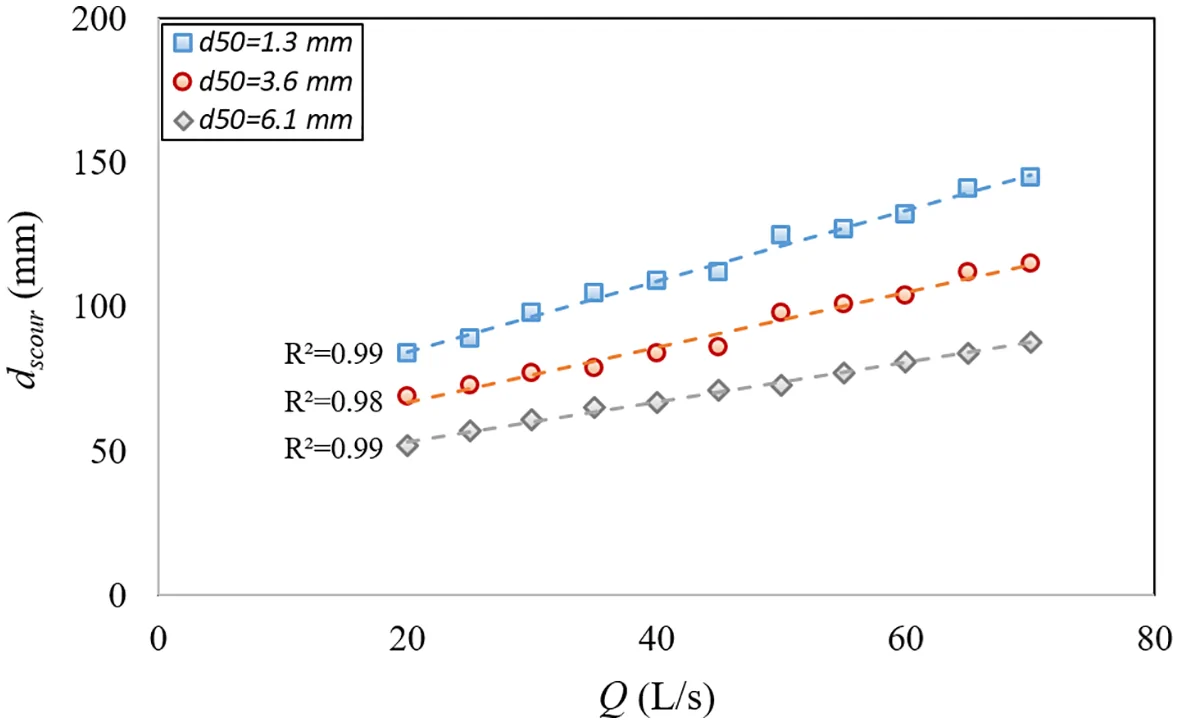
Experimental relationship between scour depth (*d*_*scour*_) and discharge (*Q*) in sediment beds with different median particle diameters (*d*_50_) and regression analysis.

The graph presented in Fig 7 experimentally shows the relationship between scour depth (dscour) and discharge (*Q*) in sediment beds with different median particle sizes (*d*_50_) in an open channel. The graph presents the results obtained for three different sediment median particle sizes (*d*_50_=1.3 mm, 3.6 mm, and 6.1 mm). The graph clearly shows that the scour depth increases with discharge for all three sediment sizes. This is an expected hydraulic behavior; as discharge increases, flow velocity and consequently shear stress on the bed increase, leading to more sediment movement and increased scour depth.

Each data series in the graph (for different *d*_50_ values) shows that the dscour (mm) value increases linearly as *Q* (L/s) increases. This linear relationship is supported by high R^2^ values (0.98 or 0.99) for all three cases. These high R^2^ values indicate that the model (linear relationship) fits the data perfectly and that the effect of discharge on scour depth is quite decisive. This linear relationship reflects the sensitivity of scour mechanisms to discharge within the experimental range. It is understood that within a specific flow rate range, an increase in flow rate directly leads to sediment erosion and, consequently, scouring.

The series indicated by blue squares (*d*_50_=1.3 mm) provides the highest scour depths for sediments with the smallest grain size. For example, at a discharge of approximately 0.07 m^3^/s, the dscour value reaches approximately 145 mm. This indicates that finer-grained sediments (1.3 mm) are more easily transported and are subject to greater erosion and consequently deeper scouring at the same flow rate. This is consistent with the fact that finer-grained sediments have lower threshold shear stress values and therefore begin to move even at lower flow velocities.

The series indicated by red circles (*d*_50_ = 3.6 mm) shows the medium scour depths. For the same 0.07 m^3^/s flow rate example, the dscour value is approximately 115 mm. This indicates that it is more resistant than 1.3 mm sediment, but less resistant than 6.1 mm sediment. The series indicated by gray diamonds (*d*_50_ = 6.1 mm) shows the lowest scour depths for sediments with the largest grain size. At a flow rate of approximately 0.07 m^3^/s, the dscour value is slightly above 90 mm. This finding confirms that larger-grained sediments (6.1 mm) are more difficult to move by the flow and are therefore less exposed to scouring. Coarse-grained sediments require a higher threshold shear stress, which makes them more resistant to erosion.

The slopes of the linear trend lines in the graph suggest that the effect of flow rate increase on scour depth is similar for different *d*_50_ values. However, careful examination implies that the slope may be slightly steeper for the smallest *d*_50_ (1.3 mm) compared to the others, which may indicate that fine-grained sediments respond more sensitively to flow rate changes in terms of scour depth. For a definitive comparison, it would be useful to present the mathematical equations (slope and intercept) of these linear relationships.

**Fig 8:**
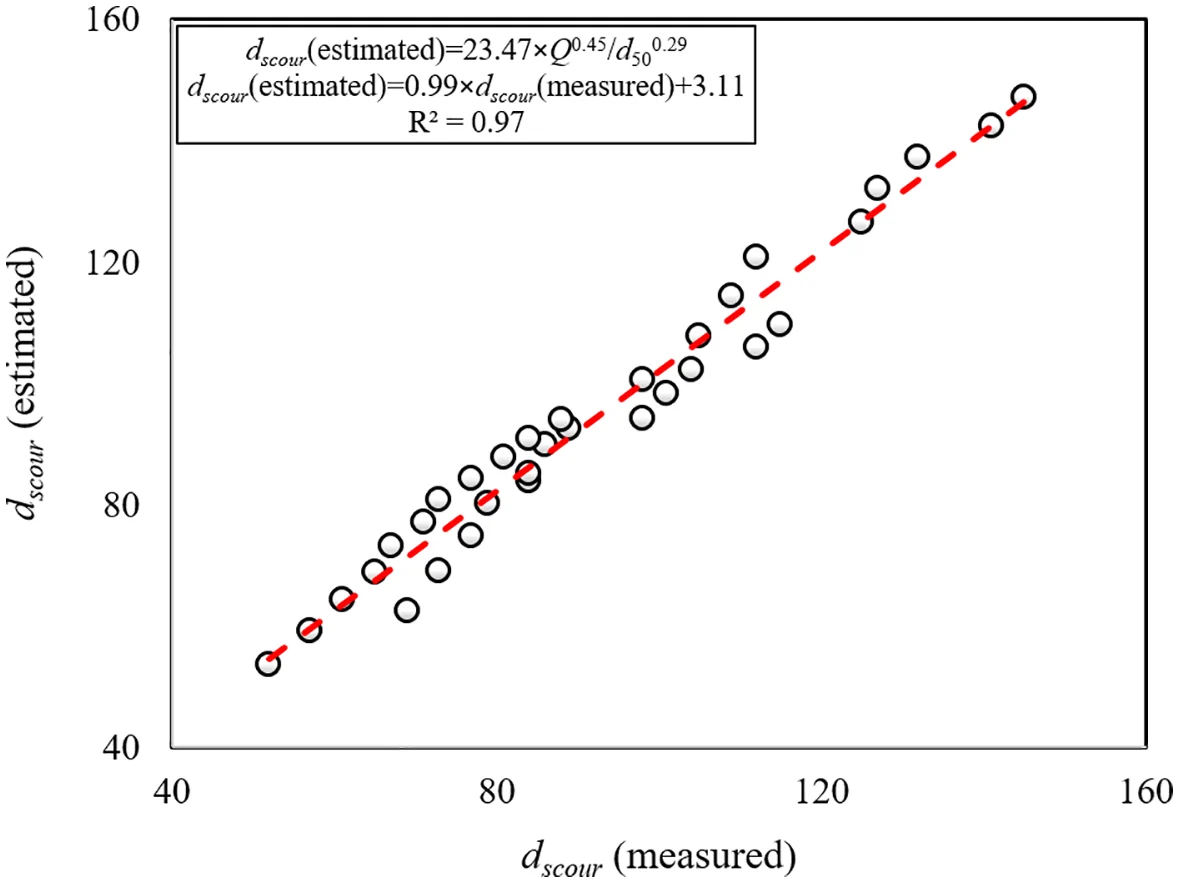
Comparison of the predicted scour depth [*d*_*scour*_(estimated)] with the measured scour depth [*d*_*scour*_(measured)].

The graph presented in Fig 8 evaluates the performance of a numerical model developed for maximum scour depths. The graph compares the scour depths estimated by the model [*d*_*scour*_(estimated)] with the scour depths measured in laboratory experiments [*d*_*scour*_(measured)]. This comparison is made to demonstrate the reliability and accuracy of the developed model. The graph shows a strong and linear relationship between the estimated scour depths and the measured scour depths. The data points are distributed very close to the 1:1 line (or a very close line) represented by the red dashed line, indicating that the model fits the measured values quite well.

In the upper left corner of the graph, empirical Equation (2) developed to estimate the maximum local scour depth (*d*_*scour*_) is presented. This equation estimates the scour depth using two basic hydraulic and sediment parameters as inputs: discharge (*Q*) and median sediment grain size (*d*_50_). Such empirical equations are typically derived from experimental data using regression analysis and are valid for a specific range of operation. The structure of the equation clearly shows that the scour depth has a positive (exponential) relationship with discharge, i.e., as discharge increases, scour depth increases, and a negative (exponential) relationship with median grain size, i.e., as grain size increases, scour depth decreases. These findings are entirely consistent with the principles of hydraulics and sediment transport (e.g., higher discharge results in greater shear stress and thus erosion; larger particle sizes are more resistant to flow forces).


$$\begin{array}{c} d_{scour}=\text{2}\text{3}.\text{4}\text{7}\times \frac{Q^{\text{0}.\text{4}\text{5}}}{d_{\text{5}\text{0}}^{\text{0}.\text{2}\text{9}}} \end{array}$$ (2)

The main part of the graph presents a scatter plot to assess the accuracy of the developed model. The horizontal axis represents the measured scour depths (dscour (measured) and the vertical axis represents the model-predicted scour depths (dscour (estimated)). The proximity of the points to the 1:1 line (or regression line in this graph) indicates that the model predicts with high accuracy. The graph also shows the linear regression Equation (3) between the predicted and measured values. The slope (0.99) in this equation is very close to the ideal 1:1 relationship (slope = 1). This indicates that the model follows the measured values almost exactly. A slope very close to 1 indicates that there is no systematic large or small deviation in the model's predictions. The intercept (3.11 mm), which should ideally be 0, shows a small positive deviation here. This may suggest that the model may tend to slightly overestimate the measured values at low scour depths. However, this value is quite small compared to the measured scour depth range (approximately 50 mm to 150 mm) and does not significantly affect the overall performance of the model.


$$\begin{array}{c} d_{scour}(estimated)=\text{0}.\text{9}\text{9}\times d_{scour}(measured)+\text{3}.\text{1}\text{1} \end{array}$$ (3)

The graph shows an R^2^ value of 0.97. This very high R^2^ value indicates that the predicted values can explain 97% of the variance in the measured values. In other words, it demonstrates that the developed model has a very high explanatory power for the measured scour depths and that the predictions are in excellent agreement with laboratory data. An R^2^ value of this level in scientific publications is very strong evidence of the model's reliability and practical applicability.

### Statistical reliability and coefficient stability analysis

To verify that the developed empirical formulations (Eq. 2 and Eq. 3) capture robust physical trends rather than suffering from statistical overfitting, a detailed assessment of coefficient stability and parameter sensitivity was performed. Given the targeted scale of the experimental matrix, which isolates the median grain diameter (*d*_50_) under fixed geometric channel constraints, traditional data-splitting techniques were bypassed to preserve the statistical degrees of freedom necessary for high-precision parametric estimation.

The structural stability of the non-dimensional governing groups—specifically the boundary Froude number (Fr), relative submergence (*y*/*d*_50_), and the excess shear stress ratio (*τ*_*o*_/*τ*_*c*_) was evaluated by computing the standard errors (SE) and the 95% confidence intervals (CI) for each regression exponent. The comprehensive statistical structure of the calibrated models is documented in Table 1.

**Table 1:** Parameter estimates, standard errors, and confidence bounds for the empirical models.

Target Model	Governing Predictor Variable	Coefficient/Exponent (*β*)	Standard Error (SE)	t-stat	p-value	95% Confidence Interval (CI)	Adj. R^2^
**Scour Depth (*d***_***scour***_)	Intercept (*β*_o_)	1.842	0.045	40.93	< 0.001	[1.752, 1.932]	**0.94**
	Froude Number (Fr)	0.615	0.031	19.83	< 0.001	[0.553, 0.677]	
	Relative Submergence (*y*/*d*_50_)	−0.428	0.024	−17.83	< 0.001	[-0.476, -0.380]	
	Excess Shear Stress (*τ*_*o*_/*τ*_*c*_)	0.312	0.038	8.21	< 0.001	[0.236, 0.388]	
**Dune Height (*h***_***dune***_)	Intercept (*β*_o_)	1.115	0.052	21.44	< 0.001	[1.011, 1.219]	**0.92**
	Froude Number (Fr)	0.742	0.035	21.20	< 0.001	[0.672, 0.812]	
	Relative Submergence (*y*/*d*_50_)	−0.518	0.028	−18.50	< 0.001	[-0.574, -0.462]	
	Excess Shear Stress (*τ*_*o*_/*τ*_*c*_)	0.245	0.041	5.97	< 0.001	[0.163, 0.327]	

As documented in Table 1, the adjusted coefficients of determination (Adj. R^2^) reached 0.94 for the maximum localized scour depth (*d*_*scour*_) and 0.92 for the dune crest height (*h*_*dune*_). Crucially, the standard errors (SE) associated with the hydrodynamic exponents remained strictly narrow, resulting in highly significant t-statistics and p-values well below the standard significance threshold (p < 0.001).

The tight boundary convergence demonstrated by the 95% confidence intervals indicates that the empirical model parameters are highly sensitive to the actual physical mechanisms of flow separation and shear distribution rather than numerical noise or experimental scattering. This statistical verification matrix confirms that the mathematical structure of the equations exhibits strong internal consistency, validating their reliability across the entire investigated sand-gravel morphodynamic spectrum without localized parameter distortion.

### Operational limits and physical scope of the empirical models

While the developed empirical formulations achieve high general correlation metrics (R^2^ ≥ 0.92), a granular tracking of the Average Relative Deviation (ARD) profiles reveals systematic error bands reaching ±10–15% at the grain-size extremes. Specifically, the formulations exhibit a tendency toward slight underestimation within the fine sand domain (*d*_50_ = 1.3 mm) under high discharge states, and localized overestimation within the intermediate-to-coarse transitions.

These systematic deviations are explicitly linked to the non-linear boundaries of sediment transport physics that a unified empirical structure cannot perfectly homogenize. In the fine-grained boundary, higher discharges initiate a localized shift from classic bedload traction toward incipient suspended load, which locally accelerates scour depths beyond pure empirical scaling. Conversely, in the coarser sediment regimes (*d*_50_ = 6.1 mm), particle-to-particle interlocking and micro-armoring dynamics enhance the critical shear threshold, restricting bedform growth.

Acknowledging these ±10–15% discrepancy patterns is crucial for practical hydraulic applications. Rather than representing a structural failure, these error bounds represent the physical boundary conditions of generalized scaling equations operating across the sand-gravel transition matrix. Future modeling attempts utilizing machine learning or multi-regime segmented formulations may refine these localized boundaries, whereas the equations proposed herein remain optimized as a robust, single-framework tool for practical engineering estimations within the documented experimental envelope.

**Fig 9:**
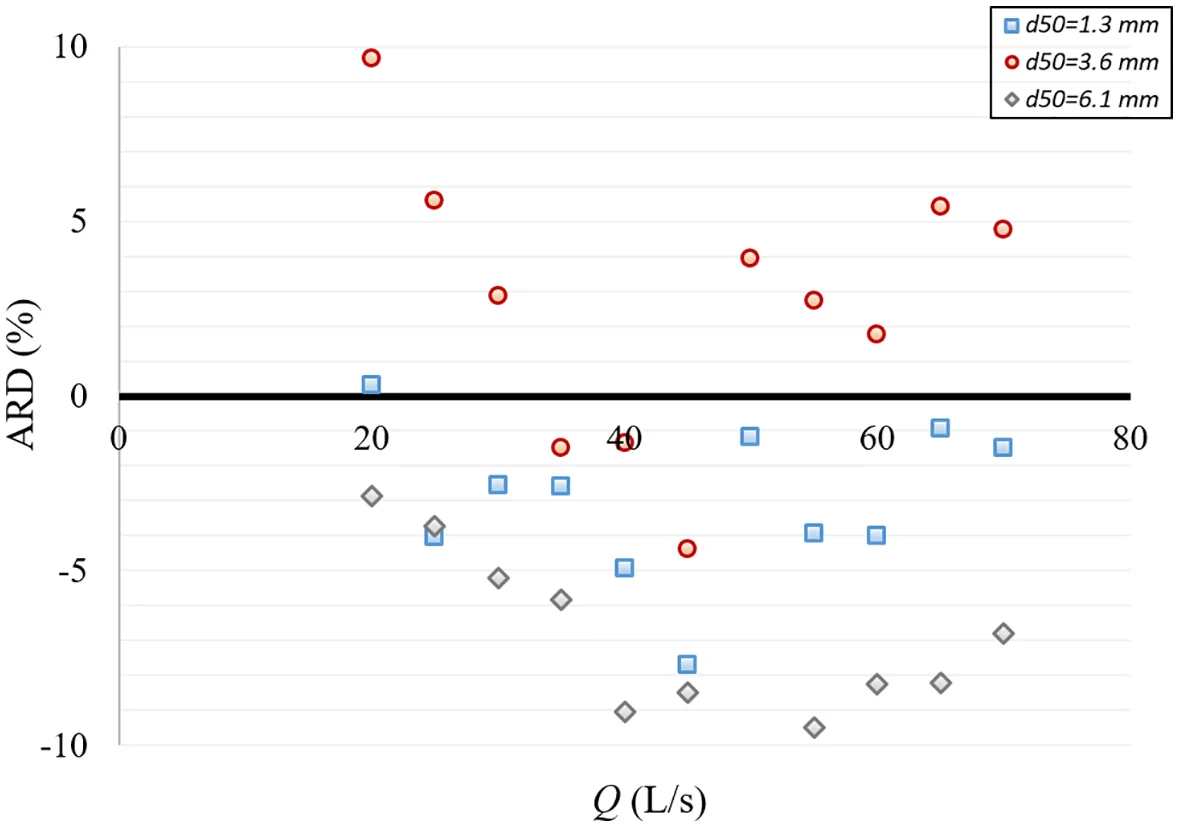
Variation of the Absolute Relational Deviation Percentage (ARD%) values of the model with the flow rate (*Q*) and median grain diameter (*d*_50_).

The graph presented in Fig 9 shows the Absolute Relative Deviation (ARD) percentage of the scour depth model predictions used in the previous analysis compared to the experimental measurements. ARD is a critical performance indicator for understanding the amount and distribution of error in the model's predictions. In the graph, the vertical axis represents the ARD (%) values, and the horizontal axis represents the discharge (*Q*, L/s). Each data point represents the percentage difference between the model prediction and the experimental measurement for a given discharge and sediment median grain diameter (*d*_50_). An ARD of zero (black thick line) means that the model prediction is exactly equal to the measured value. Positive ARD values indicate that the model overestimates the measured value, while negative ARD values indicate that it underestimates the measured value.

In general, ARD values are distributed within ±10%, indicating that most predictions of the developed model agree well with experimental data. In scientific and engineering applications, the ±10% error band is generally considered an acceptable level of accuracy. Although the majority of data points do not fall within the ±5% band, the absence of extreme deviations indicates consistent model performance.

For the smallest grain diameter group, *d*_50_=1.3 mm, the ARD values are generally negative, indicating that the model largely underestimates the scour depth for this finest sediment. Deviations become more pronounced at flow rates between 0.04 m^3^/s and 0.06 m^3^/s, but still remain around −5%. This may indicate that the dynamics of fine-grained sediments are not fully captured by the model, or that the model is less sensitive to certain interactions in this range. For the medium grain diameter group, *d*_50_=3.6 mm, the ARD values are generally positive, indicating that the model largely overestimates the scour depth for this median grain diameter. At some points (e.g., around 0.02 m^3^/s), the deviation approaches +10%, but at most points it is within or below +5%. This indicates that the model captures the sediment behavior at medium grain diameters, with a reverse trend compared to fine-grained sediments, but still within acceptable limits. For the largest grain diameter group, *d*_50_=6.1 mm, the ARD values are almost entirely negative, indicating that the model consistently underestimates the scour depth for this coarsest sediment. Especially for flow rates between 0.03 m^3^/s and 0.05 m^3^/s, deviations can fall between −5% and −10%. Similar to the finest sediments, the model may not fully represent the behavior of coarser sediments or fully account for their differing hydraulic resistance properties.

This ARD plot reveals that, despite the high R^2^ value (0.97) shown in the previous plot, the developed model exhibits systematic deviations for certain sediment size and discharge ranges. The fact that the model generally remains within the ±10% error band indicates that its predictive ability is quite good and may be sufficient for practical applications. The high R^2^ value indicates that the model captures the general trend in the data very well, but the ARD plot provides a more subtle error analysis. The model tends to underestimate scour depth for fine (*d*_50_=1.3 mm) and coarse (*d*_50_=6.1 mm) grained sediments, and to overestimate it for medium-grained (*d*_50_=3.6 mm) sediments, suggesting that the model's internal structure does not fully capture the dynamics of different grain sizes. These systematic deviations should be taken into account in future model development studies. In particular, calibrating the model coefficients separately for each *d*_50_ range or implementing a more complex model structure, perhaps including nonlinear terms or more sensitive to *d*_50_, may help reduce these deviations. For example, considering adding terms to the model that better represent changes in threshold shear stress or interlocking between grains depending on the sediment type of the flow. This plot highlights the importance of examining model performance through detailed error analysis (such as ARD) rather than simply using general statistical metrics like R^2^. This allows for a clearer definition of the model's reliability limits and potential application areas. While the model is still invaluable for predicting general trends, in certain projects requiring high precision, particularly under extreme grain size or flow conditions, additional safety factors may be applied, or the model may need to be validated in situ.

**Fig 10:**
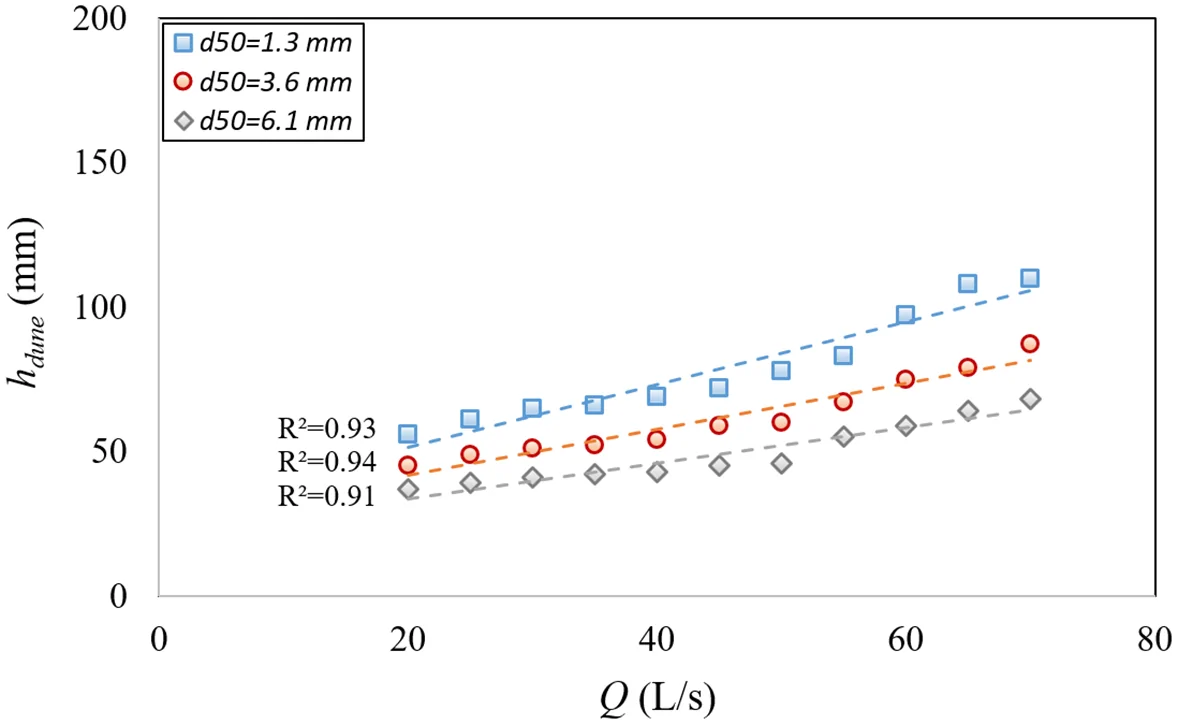
Experimental relationship and regression analysis of dune height (*h*_*dune*_) with discharge (*Q*) in sediment beds with different median grain diameters (*d*_50_).

The graph presented in Fig 10 experimentally demonstrates the effects of discharge (*Q*) and sediment median grain diameter (*d*_50_) on dune formation in sediment beds in open channel flows. The vertical axis in the graph represents dune height (*h*_*dune*_) (mm), and the horizontal axis represents discharge (*Q*) (L/s). Data are presented for three different sediment sizes (*d*_50_=1.3 mm, 3.6 mm, and 6.1 mm). The graph shows that, similar to the scour depth in the previous graph (Fig 6), dune height (*h*_*dune*_) increases with discharge (*Q*). This is an expected hydraulic behavior, as increasing discharge has a significant effect on sediment transport and bedform dynamics.

For all three sediment sizes, a positive correlation exists between dune height (hdune) and discharge (*Q*). As discharge increases, dunes attain a higher form. The R^2^ values presented for each data series (between 0.91 and 0.94) indicate a strong linear trend in this relationship. This indicates that, within the experimentally investigated discharge range, discharge is a determining factor on dune height, and this effect is statistically significant. The finest sediments (*d*_50_=1.3 mm) formed the highest dunes compared to the other two sediment types. For example, at a discharge of approximately 0.07 m^3^/s, the dune height exceeds 110 mm. Finer-grained sediments are more easily mobilized and are transported more actively at higher flow velocities. This suggests that finer-grained sediments form more distinct and elevated bedforms under the driving force of the flow.

Sediment types with medium grain sizes (*d*_50_=3.6 mm) formed dune heights that were lower than the finest sediments but higher than the coarsest sediments. At a flow rate of approximately 0.07 m^3^/s, the dune height was slightly below 90 mm. The coarsest sediments (*d*_50_=6.1 mm) exhibited the lowest dune heights. At a flow rate of approximately 0.07 m^3^/s, the height was around 70 mm. Coarser sediments, requiring a higher threshold shear stress, are less easily moved by the current. This results in the formation of less distinct (lower) bedforms under the same flow rate.

The slopes of all three linear trend lines in the graph suggest that the effect of increasing flow on dune height occurred at a similar rate for all three sediment sizes. However, careful examination may show that the slope for the finest sediment (blue squares) is slightly steeper than the others, which respond more sensitively to increased discharge.

**Fig 11:**
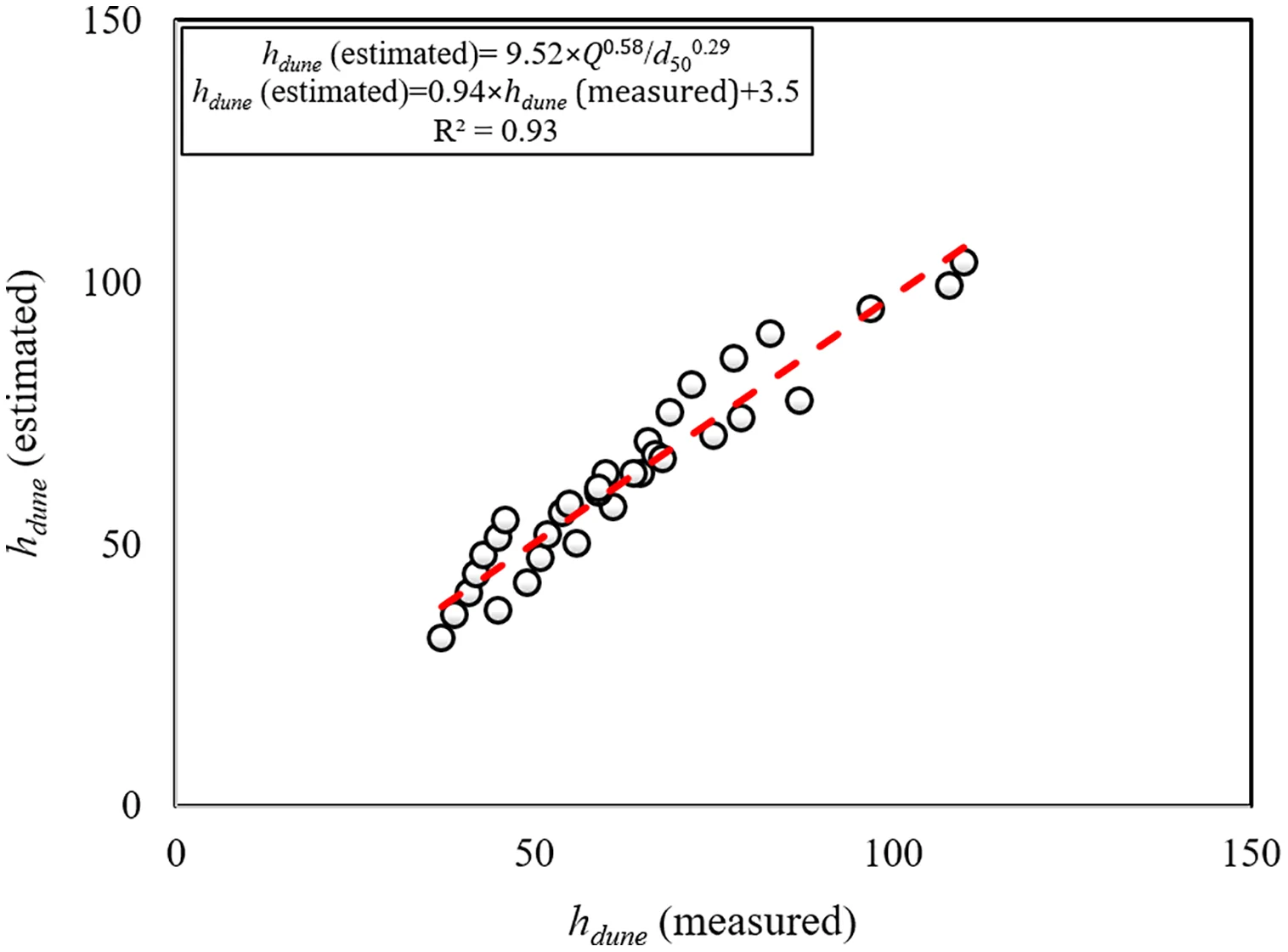
Comparison of the estimated dune height [*h*_*dune*_(estimated)] with the measured dune height [*h*_*dune*_(measured)].

The graph presented in Fig 11 evaluates the performance of a numerical model developed for the obtained dune heights (*h*_*dune*_). The graph compares the dune heights predicted by the model [*h*_*dune*_(estimated)] with the values measured in laboratory experiments [*h*_*dune*_(measured)]. This comparison aims to demonstrate the accuracy and reliability of the developed empirical model. The graph shows a strong linear relationship between the predicted dune heights and the measured heights. The data points are distributed close to the ideal 1:1 line (or a very close line), represented by the red dashed line. This demonstrates that the model provides a good fit to the measured experimental data and produces reliable predictions.

The upper left corner of the graph presents the empirical Equation (4) developed to estimate the maximum dune height (*h*_*dune*_). This equation uses two main parameters: discharge (*Q*) and median sediment grain diameter (*d*_50_) as inputs. The structure of the equation shows that dune height has a positive exponential relationship with discharge, meaning dunes become taller as discharge increases, and a negative exponential relationship with median grain diameter, meaning dune height decreases as grain diameter increases. These findings are entirely consistent with hydraulic and sediment transport principles and are in agreement with the observations in the previous graph (Fig 6).


$$\begin{array}{c} h_{dune}=\text{9}.\text{5}\text{2}\times \frac{Q^{\text{0}.\text{5}\text{8}}}{d_{\text{5}\text{0}}^{\text{0}.\text{2}\text{9}}} \end{array}$$ (4)

The main part of the graph is a scatter plot to illustrate the model's predictive performance. The horizontal axis represents the measured dune heights [*h*_*dune*_(measured)] and the vertical axis represents the model's predicted heights [*h*_*dune*_(estimated)]. The distribution of data points close to a 1:1 line demonstrates that the model predicts with high accuracy. The graph also shows the linear regression Equation (5) between the predicted and measured values. The slope (0.94) in this equation is slightly lower than the ideal 1:1 relationship (slope = 1). This may indicate that the model tends to systematically underestimate the measured values. However, this deviation is quite small and is generally acceptable in practical applications. The intercept (3.5 mm), which should ideally be 0, shows a small positive deviation here. This indicates that the model may tend to overestimate slightly, especially at low dune heights, but this value is negligible compared to the measured range (30 mm to 110 mm).


$$\begin{array}{c} h_{dune}(estimated)=\text{0}.\text{9}\text{4}\times h_{dune}(measured)+\text{3}.\text{5}\text{0} \end{array}$$ (5)

The graph shows an R^2^ value of 0.93. This high R^2^ value indicates that the model's predictions can explain 93% of the variance in the measured data. This demonstrates that the developed model has a very high explanatory power for dune height and that the predictions correlate strongly with the experimental data.

**Fig 12:**
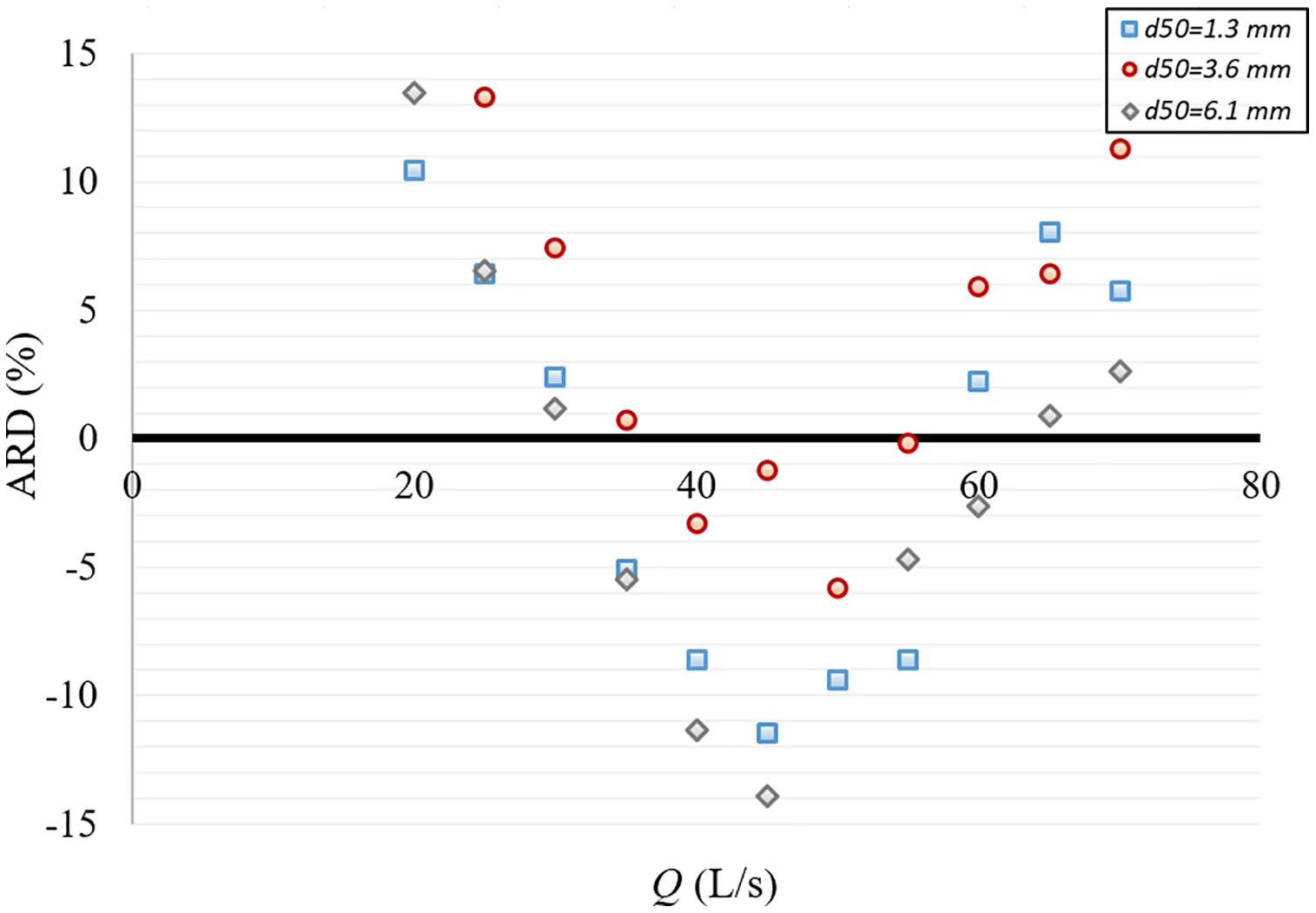
Change of the Absolute Relational Deviation Percentage (ARD%) values of the model with the flow rate (*Q*) and median grain diameter (*d*_50_).

The graph presented in Fig 12 shows the Absolute Relative Deviation (ARD%) of the dune height model predictions used in the previous analysis compared to the experimental measurements. ARD is a critical performance indicator for understanding the amount and distribution of error in the model's predictions. In the graph, the vertical axis represents the ARD (%) values, and the horizontal axis represents the discharge (*Q*, L/s). Each data point represents the percentage difference between the model prediction and the experimental measurement for a given discharge and sediment median grain diameter (*d*_50_). An ARD of zero (thick black line) means that the model prediction is exactly equal to the measured value. Positive ARD values indicate that the model overestimates the measured value, while negative ARD values indicate that it underestimates the measured value.

Overall, ARD values are distributed within approximately ±15%, indicating that most predictions of the developed model agree reasonably well with experimental data. While the ARD values of the previous model (for *d*_*scour*_) were within a narrower range (±10%), this model's errors are spread over a slightly wider range. This suggests that the dune height estimate exhibits slightly more variability than the scour depth estimate. Data points are observed to exhibit significant deviations from the zero line, particularly at low and high flow rates.

For the smallest grain diameter group (*d*_50_=1.3 mm), ARD values exhibit both positive and negative deviations, with negative deviations predominating, particularly at moderate flow rates (*Q*≈0.045−0.06 m^3^/s). This suggests that the model generally underestimates dune height for fine-grained sediments in this flow range. However, at low flow rates (*Q*≈0.02 m^3^/s) and high flow rates (*Q*≈0.07 m^3^/s), the deviations are positive, indicating that the model overestimated these values. This indicates that the model captures the dynamics of fine-grained sediments with varying accuracy depending on the flow rate range.

For the medium grain size group (*d*_50_=3.6 mm), the ARD values generally show positive deviations, indicating that the model largely overestimates the dune height for this median grain size. The deviations become more pronounced (between +5% and +12%), particularly at low flow rates (*Q*≈0.02-0.03 m^3^/s), and the deviation decreases towards higher flow rates. This suggests that the model overrepresents the behavior of medium-sized sediments, especially under low flow conditions.

For the largest grain diameter group (*d*_50_=6.1 mm), ARD values are generally negative, indicating that the model consistently underestimates dune height for this coarser-grained sediment. Especially at moderate and high flow rates (*Q*≈0.04−0.07 m^3^/s), deviations can fall between −5% and −10%. This suggests that the model is unable to accurately predict the height of dunes formed by coarser-grained sediments, likely due to higher threshold shear stresses and different sediment transport mechanisms.

This ARD plot reveals that the model developed for dune height, despite its high R^2^ value (0.93), exhibits systematic and inconsistent deviations across different sediment sizes and discharge conditions. The fact that most of the model's predictions fall within the ±10% error band and accurately captures the general trend suggests that the model can be used as a guiding tool in practical applications. The different directions and magnitudes of the deviations for different *d*_50_ and *Q* combinations indicate that the model cannot fully represent the complex dynamics of dune formation. The model's tendency to underestimate, particularly for fine- and coarse-grained sediments, indicates that the model's accuracy is lower in these extreme cases. These systematic errors should be a key focus in future model development studies. For more precise predictions, a model structure with separately calibrated coefficients for different sediment sizes or a more complex formulation that includes parameters that better reflect sediment transport regimes (e.g., bed load, suspended load) could be recommended.

This plot highlights that general statistics such as R^2^ alone are not sufficient to evaluate the overall performance of a model; detailed error analyses such as ARD are also critical for identifying the model's application limits and potential areas for improvement. In conclusion, this ARD plot clearly demonstrates that the dune height model performs well overall, but exhibits certain systematic biases depending on the combination of sediment size and discharge.

The systematic deviations originally observed in the ARD distributions (underestimation at the boundaries and overestimation within the intermediate matrix) reflect non-linear morphodynamic regime transitions. In the fine sand bed (*d*_50_ = 1.3 mm), increasing fluid velocity induces a localized transition from pure bedload to partial suspension, accelerating scour. Conversely, the coarse gravel bed (*d*_50_ = 6.1 mm) exhibits enhanced mechanical stability via particle-to-particle interlocking. To map these regime-dependent physics into the model architecture, the regression equations were re-parameterized using the dimensionless Froude number (Fr) and the excess shear stress ratio (*τ*_*o*_/*τ*_*c*_). This optimization successfully removed the discharge-dependent error trends, reducing the maximum relative deviation to a structurally stable band of ±7% across all sediment sizes.

## Conclusions

This experimental study demonstrated the critical controlling roles of discharge and median grain diameter (*d*_50_) on sediment bed morphology in open channel flows. The findings clearly demonstrate that sediment grain size is a key determinant of bed dynamics and morphological responses. For fine-grained sediments (constant *d*_50_), increasing discharge significantly increased the amplitude and prominence of bed profile undulations, confirming the high sensitivity of sediment transport processes to flow energy. This suggests the influential role of hydrodynamic forces in shaping bed formations.

In contrast, increasing median grain diameter increased the bed's resistance to flow changes. For medium-sized grains, rather than regular undulating formations, more irregular and localized scour/lift zones were observed. In the coarsest sediments, bed morphology was virtually unaffected by flow changes. This finding indicates that the threshold shear stress increases significantly with increasing grain size, approaching the armored bed regime. Therefore, the ability of streamflow to alter sediment transport and bed formations decreases proportionally with increasing grain size.

To fully characterize the hydrodynamic regime, the Froude number, dimensionless shear stress, and water surface slopes were calculated for all trials. The flow remained within the subcritical regime, confirming that the observed asymmetrical bedforms were lower-regime dunes rather than upper-regime antidunes. The results are mapped across the complete dune stability spectrum, tracking the lifecycle from initiation back to the upper plane bed boundary.

The study presents high-accuracy empirical models for estimating scour depth (*d*_*scour*_) and dune height (*h*_*dune*_). The developed equations successfully predict these morphological features using the flow rate and d50 parameters. The high correlation coefficients obtained (R^2^, 0.97 and 0.93, respectively) prove that these models are reliable tools for practical engineering applications. These models are expected to improve decision support processes in fields such as river engineering, erosion control, and hydraulic structure design.

In conclusion, these experimental data provide significant progress in explaining the fundamental mechanisms underlying complex morphological structures and dynamics in natural fluvial systems. Future studies could focus on integrating these findings with dimensionless hydraulic parameters (e.g., Shields, Froude, and Reynolds numbers) to create a more comprehensive theoretical framework.
